# Unexpected combined effects of NADH dehydrogenase subunit-2 237 Leu/Met polymorphism and green tea consumption on renal function in male Japanese health check-up examinees: a cross-sectional study

**DOI:** 10.1186/1477-5751-12-17

**Published:** 2013-11-20

**Authors:** Akatsuki Kokaze, Mamoru Ishikawa, Naomi Matsunaga, Kanae Karita, Masao Yoshida, Tadahiro Ohtsu, Hirotaka Ochiai, Takako Shirasawa, Hinako Nanri, Hiromi Hoshino, Yutaka Takashima

**Affiliations:** 1 Department of Public Health, Showa University School of Medicine, 1-5-8 Hatanodai, Shinagawa-ku, Tokyo 142-8555, Japan; 2 Department of Public Health, Kyorin University School of Medicine, 6-20-2 Shinkawa, Mitaka-shi, Tokyo 181-8611, Japan; 3 Mito Red Cross Hospital, 3-12-48 Sannomaru, Mito-shi, Ibaraki 310-0011, Japan

**Keywords:** Chronic kidney disease, Estimated glomerular filtration rate, Green tea consumption, NADH dehydrogenase, Polymorphism

## Abstract

**Background:**

NADH dehydrogenase subunit-2 237 leucine/methionine (ND2-237 Leu/Met) polymorphism is associated with longevity in Japanese. A previous study has shown that ND2-237 Leu/Met polymorphism modulates the effects of green tea consumption on risk of hypertension. For men with ND2-237Leu, habitual green tea consumption may reduce the risk of hypertension. Moreover, there is a combined effect of ND2-237 Leu/Met polymorphism and alcohol consumption on risk of mildly decreased estimated glomerular filtration rate (eGFR) (<90 ml/min/1.73 m^2^). Several beneficial effects of green tea on the kidney have been reported. The objective of this study was to investigate whether ND2-237 Leu/Met polymorphism modifies the effects of green tea consumption on risk of mildly decreased eGFR in male Japanese health check-up examinees.

**Results:**

For ND2-237Leu genotypic men, after adjustment for confounding factors, green tea consumption may increase the risk of mildly decreased eGFR (*P* for trend = 0.016). The adjusted odds ratio (OR) for mildly decreased eGFR was significantly higher in subjects with ND2-237Leu who consume ≥6 cups of green tea per day than those who consume ≤1 cup of green tea per day (adjusted OR = 5.647, 95% confidence interval: 1.528-20.88, *P* = 0.009). On the other hand, for ND2-237Met genotypic men, green tea consumption does not appear to determine the risk of mildly decreased eGFR.

**Conclusion:**

The present results suggest that ND2-237 Leu/Met polymorphism unexpectedly modifies the effects of green tea consumption on eGFR and the risk of mildly decreased eGFR in male Japanese subjects.

## Background

Green tea consumption contributes to a reduction in the risk of cardiovascular disease and some types of malignancy, as well as to the promotion of oral health and other physiological functions [1]. Several beneficial effects of green tea on the kidney have also been reported [2-11]. In experimental animal models, green tea extract (GTE) exhibits protective effects on renal disorder [2-9]. *In vitro* experiments have shown that GTE exerts protective effects against oxidative injury in human mesangial cells [10]. A case–control study reported that green tea consumption is associated with a decreased risk of renal cell carcinoma in the Chinese population [11].

Mitochondrial DNA cytosine/adenine polymorphism, which is also recognized as NADH dehydrogenase subunit-2 237 leucine/methionine (ND2-237 Leu/Met) polymorphism, is associated with longevity in Japanese [12]. The frequency of the ND2-237Met genotype is significantly higher in Japanese centenarians than in the general population. On the other hand, Japanese individuals with ND2-237Leu are more susceptible to adult-onset diseases, such as hypertension [13], diabetes [14], myocardial infarction [15,16] and cerebrovascular disorders [17], than those with ND2-237Met. Our previous cross-sectional study showed that ND2-237 Leu/Met polymorphism modulates the effects of green tea consumption on the risk of hypertension [18]. For men with ND2-237Leu, green tea consumption may reduce the risk of hypertension. We also reported the joint effects of this polymorphism and alcohol consumption on estimated glomerular filtration rate (eGFR) [19]. For ND2-237Met genotypic men, alcohol consumption may ameliorate eGFR. Both reports [18,19] demonstrated the positive effects of green tea consumption on renal function, at least in either genotype of ND2-237 Leu/Met polymorphism.

The aim of this study was to investigate whether ND2-237 Leu/Met polymorphism modulates the effects of green tea consumption on kidney function in male Japanese health check-up examinees.

## Results

No significant differences in clinical characteristics were observed between the ND2-237Leu and ND2-237Met genotypes (Table 1). However, although differences did not reach significance, serum creatinine levels were higher and eGFR was lower in men with ND2-237Leu than in those with ND2-237Met (*P* = 0.090 and *P* = 0.066, respectively). Chi-squared test showed no significant differences in green tea consumption between the ND2-237 Leu/Met genotypes.

**Table 1:** Clinical characteristics of study subjects by ND2-237 Leu/Met genotype

	**ND2-237Leu**	**ND2-237Met**	***P *** **value**
	**N = 239**	**N = 155**	
Age (y)	54.3 ± 7.8	53.2 ± 7.8	0.171
Body mass index (kg/m^2^)	23.3 ± 2.8	23.5 ± 2.6	0.461
Systolic blood pressure (mmHg)	125.8 ± 15.9	125.7 ± 14.1	0.940
Diastolic blood pressure (mmHg)	74.0 ± 10.7	73.8 ± 9.1	0.823
Serum total cholesterol (mg/dl)	203.5 ± 34.3	202.1 ± 31.8	0.672
Serum HDL cholesterol (mg/dl)	54.6 ± 13.6	56.3 ± 16.2	0.269
Serum triglyceride (mg/dl)	136.7 ± 91.1	139.5 ± 90.8	0.766
Fasting plasma glucose (mg/dl)	97.2 ± 9.4	97.6 ± 9.7	0.672
Serum uric acid (mg/dl)	5.98 ± 1.21	5.93 ± 1.21	0.673
Blood urea nitrogen (mg/dl)	15.8 ± 3.7	15.2 ± 3.4	0.143
Serum creatinine (mg/dl)	0.817 ± 0.117	0.797 ± 0.111	0.090
eGFR (ml/min/1.73 m^2^)	79.1 ± 13.1	81.5 ± 12.4	0.066
Renal function (eGFR ≥ 90/ 90 > eGFR ≥ 60/ eGFR < 60) (%)	17.6/77.8/4.6	25.2/72.2/2.6	0.133
Antihypertensive medications (%)	18.8	12.9	0.122
Green tea consumption (≤1 cups per day/2-3 cups per day/4-5 cups per day/≥6 cups per day) (%)	26.8/30.1/21.8/21.3	27.7/33.6/20.0/18.7	0.840
Alcohol consumption (daily/occasionally/non-) (%)	46.4/35.2/18.4	47.7/38.7/13.6	0.426
Smoking status (non- or ex-/ 1–20 cigarettes per day/ >20 cigarettes per day) (%)	59.4/29.7/10.9	59.4/25.8/14.8	0.429
Coffee consumption (≤1 cup per day/2-3 cups per day/≥4 cups per day) (%)	61.1/29.7/9.2	55.5/32.9/11.6	0.510

*HDL*: high-density lipoprotein. *eGFR*: estimated glomerular filtration rate. Age, body mass index, systolic blood pressure, diastolic blood pressure, serum total cholesterol levels, serum *HDL* cholesterol levels, serum triglyceride levels, fasting plasma glucose levels, serum uric acid levels, blood urea nitrogen levels, serum creatinine levels and *eGFR* are given as means ± *S.D*. For renal function, antihypertensive medications, green tea consumption, alcohol consumption, smoking status and coffee consumption, *P* values were calculated by chi-squared test. All *P* values depict significance of differences between ND2-237Leu and ND2-237Met.

For both ND2-237Leu and ND2-237Met genotypic men, the frequency of green tea consumption was negatively and significantly associated with eGFR (*P* for trend = 0.031 and *P* for trend = 0.049, respectively) (Table 2). However, after adjustment for age and BMI, or for age, BMI, alcohol consumption, habitual smoking, coffee consumption and use of antihypertensive medication, significant associations of green tea consumption with eGFR disappeared in both genotypes.

**Table 2:** Estimated glomerular filtration rate by green tea consumption status and ND2-237 Leu/Met genotype

**Genotype and green tea consumption**	**eGFR (ml/min/1.73 m**^**2**^**)**	**eGFR**^**†**^** (ml/min/1.73 m**^**2**^**)**	**eGFR**^**‡**^** (ml/min/1.73 m**^**2**^**)**
ND2-237Leu (N = 239)			
≤1 cup per day (N = 64)	81.7 ± 1.6	80.1 ± 1.6	78.5 ± 2.0
2–3 cups per day (N = 72)	80.2 ± 1.5	79.6 ± 1.5	77.2 ± 2.0
4–5 cups per day (N = 52)	75.6 ± 1.8	77.1 ± 1.7	74.4 ± 2.1
≥6 cups per day (N = 51)	77.8 ± 1.8	79.2 ± 1.7	76.6 ± 2.2
	*P* for trend = 0.031	*P* for trend = 0.484	*P* for trend = 0.262
ND2-237Met (N = 155)			
≤1 cup per day (N = 43)	84.9 ± 1.9	84.1 ± 1.8	81.7 ± 2.4
2–3 cups per day (N = 52)	80.3 ± 1.7	79.6 ± 1.7	77.6 ± 2.3
4–5 cups per day (N = 31)	82.2 ± 2.2	82.5 ± 2.1	82.2 ± 2.6
≥6 cups per day (N = 29)	78.1 ± 2.3	80.4 ± 2.3	79.0 ± 2.8
	*P* for trend = 0.049	*P* for trend = 0.355	*P* for trend = 0.674

*eGFR*: estimated glomerular filtration rate. ^†^e*GFR* is given as least-square mean ± *S.E*. adjusted for age, body mass index; ^‡^*eGFR* is given as least-square mean ± *S.E.* adjusted for age, body mass index, habitual smoking, alcohol consumption, coffee consumption and antihypertensive medication use. Bonferroni correction for multiple comparisons was used.

For subjects with ND2-237Leu, when compared with those who consume ≤1 cup of green tea per day, the odds ratios (ORs) for mildly decreased eGFR were 1.957, 2.152 and 4.598 for consumption of 2–3, 4–5 and ≥6 cups of green tea per day, respectively (*P* for trend = 0.007) (Table 3). After controlling for age, BMI, alcohol consumption, habitual smoking, coffee consumption and use of antihypertensive medication, the positive associations between increasing frequency of green tea consumption and the risk of mildly decreased eGFR remained significant (*P* for trend = 0.016). The crude OR for mildly decreased eGFR was significantly higher in subjects with ND2-237Leu who consume ≥6 cups of green tea per day than in those who consume ≤1 cup of green tea per day (OR = 4.598, 95% confidence interval (CI): 1.445-14.63, *P* = 0.010). After the aforementioned variables were adjusted, a significant OR remained (adjusted OR = 5.647, 95% CI: 1.528-20.88, *P* = 0.009). On the other hand, the association between ND2-237Met genotype and the risk of mildly decreased eGFR does not appear to depend on green tea consumption. However, after controlling the aforementioned variables, the OR for mildly decreased eGFR was significantly higher in subjects with ND2-237Met who consume 2–3 cups of green tea per day than in those who consume ≤1 cup of green tea per day (OR = 2.943, 95% CI: 1.023-8.461, *P* = 0.045).

**Table 3:** **Odds ratios (ORs) and 95% confidence intervals (CIs) for decreased eGFR (< 90 ml/min/1.73 m**^**2**^**) by ND2-237 Leu/Met genotype and green tea consumption status**

**Genotype and green tea consumption**	**OR (95% CI)**	**Adjusted OR**^**†**^** (95% CI)**	**Adjusted OR**^**‡**^** (95% CI)**
ND2-237Leu			
≤1 cup per day	1 (reference)	1 (reference)	1 (reference)
2–3 cups per day	1.957 (0.857–4.466)	1.777 (0.748–4.223)	2.240 (0.864–5.808)
4–5 cups per day	2.152 (0.849–5.453)	1.279 (0.457–3.578)	1.509 (0.502–4.533)
≥6 cups per day	4.598 (1.445–14.63)*	3.134 (0.938–10.47)	5.647 (1.528–20.88)**
	*P* for trend = 0.007	*P* for trend = 0.064	*P* for trend = 0.016
ND2-237Met			
≤1 cup per day	1 (reference)	1 (reference)	1 (reference)
2–3 cups per day	2.250 (0.886–5.715)	2.251 (0.886–5.718)	2.943 (1.023–8.461)*
4–5 cups per day	1.540 (0.555–4.271)	1.499 (0.525–4.280)	1.136 (0.344–3.749)
≥6 cups per day	2.054 (0.687–6.143)	1.597 (0.490–5.204)	0.984 (0.260–3.721)
	*P* for trend = 0.256	*P* for trend = 0.500	*P* for trend = 0.903

*eGFR*: estimated glomerular filtration rate. ^†^*OR* adjusted for age and body mass index. ^‡^*OR* adjusted for age, body mass index, habitual smoking, alcohol consumption, coffee consumption and antihypertensive medication. *P < 0.05, **P < 0.01.

## Discussion

In the present study, we observed that ND2-237 Leu/Met polymorphism apparently modifies the effects of green tea consumption on the risk of mildly decreased eGFR in Japanese male subjects. Considering that green tea consumption contributes to human health [1], more consumption of green tea is expected to exert a desirable effect on kidney function in either genotype. However, unexpectedly, for men with ND2-237Leu, green tea drinking may increase the risk of mildly decreased eGFR. On the other hand, for those with ND2-237Met, green tea consumption does not appear to influence the risk of mildly decreased eGFR.

Certainly, in experimental animal models, there have been numerous investigations into the protective effects of GTE on renal damage [2-9]. GTE improves eGFR in streptozotocin-induced diabetic rats [2]. Green tea also prevents an increase in creatinine clearance [3]. Judging from serum creatinine levels, GTE obliterates cisplatin-induced nephrotoxicity in rats [4-6]. Moreover, GTE also exerts antiproteinuric effects on cyclosporine-induced nephrotoxicity in rats [7]. Furthermore, GTE reverses the progression of immune-mediated glomerulonephritis in mice [8]. GTE may exert these protective effects on renal damage through its antioxidant activity [2,4-9]. However, Inoue et al. reported that a diet containing high-dose green tea polyphenols disrupts renal function in both mice with dextran sulfate sodium-induced colitis and normal mice [20]. They assumed that high-dose green tea polyphenols down-regulate antioxidant enzymes, leading to kidney dysfunction. However, in human subjects, to our knowledge, there is no solid evidence reporting beneficial effects of green tea consumption on renal function. A clinical trial reported that green tea consumption does not increase eGFR in 19 healthy young adults, while coffee consumption increases it [21]. Population-based prospective studies, at least cross-sectional studies, focusing on the association between green tea consumption and renal function or risk of chronic kidney disease (CKD) are thus necessary.

NADH dehydrogenase is recognized as the major physiological and pathological site of reactive oxygen species (ROS) generation in mitochondria, and as a target of attack by ROS [22]. There may be biophysical and biochemical differences in the protection against ROS or the reduction of ROS generation between ND2-237Leu and ND2-237Met. Considering the results from experimental animal models [23,24], ND2-237Met may suppress ROS production and/or protect NADH dehydrogenase itself from ROS. Therefore, in order to determine the mechanisms responsible for the unexpected joint effects of ND2-237 Leu/Met genotypes and green tea consumption on renal function, further biophysical and biochemical investigations are necessary.

In addition to other molecular epidemiological studies [25,26] and our previous study [19], in accordance with Kidney Disease Outcomes Quality Initiative (K/DOQI) CKD classification [27], eGFR of <90 ml/min/1.73 m^2^ was defined as reduced eGFR in this cross-sectional study. In the present subjects, the prevalence of eGFR of <90 ml/min/1.73 m^2^ was 82.4% in men with ND2-237 Leu and was 74.8% in those with ND2-237 Met. A large-scale population-based epidemiological study showed that the prevalence of eGFR of <90 ml/min/1.73 m^2^ was above 80% in Japanese men aged 30–69 years [28]. Hence, the validity of the definition of reduced eGFR adopted in this study is worth further consideration.

This study has several critical limitations. First, the study sample size was insufficient. Second, in addition to information bias due to the self-reported questionnaire, selection bias is probable due to the recruiting of subjects from those visiting the hospital for regular medical check-ups, and the prevalence of moderately decreased eGFR of <60 ml/min/1.73 m^2^, generally recognized as CKD, was low among study subjects. Third, this study was a cross-sectional study, and although the study design is able to suggest causal links, it cannot determine rational causality. To overcome these limitations, a large-scale population-based prospective study is necessary. Fourth, the evaluation of green tea consumption was based on the number of cups consumed per day. Although we have used this evaluation method previously [18], we will require data on serving size, green tea brands and purity, and concentrations of GTE, such as (−)-epigallocatechin-3-gallate, and other catechins in green tea, for further investigations. Fifth, data on dietary factors or socioeconomic factors, which are reported as risk factors for CKD [29], were not collected in this study. Finally, we lacked data on proteinuria, which is an early and sensitive marker of kidney damage in various types of CKD [28]. Therefore, future research should examine proteinuria in order to preventively estimate renal damage.

## Conclusion

Despite several crucial limitations in this study, longevity-associated ND2-237 Leu/Met polymorphism appears to modulate the effects of green tea consumption on eGFR or the risk of mildly decreased eGFR in Japanese men. Surprisingly, for ND2-237Leu genotypic men, green tea intake may decrease eGFR or increase the risk of mildly decreased eGFR. From the viewpoint of preventing hypertension, green tea consumption is recommended for men with ND2-237Leu [18]. Therefore, considering that both hypertension and CKD are risk factors of cardiovascular diseases, further investigation will be necessary to establish the personalized optimum for maximizing the risk reduction and minimizing the risk increase of green tea consumption for cardiovascular diseases.

## Methods

### Subjects

Participants were recruited from among individuals visiting the Mito Red Cross Hospital for regular medical check-ups between August 1999 and August 2000. This study was conducted in accordance with the Declaration of Helsinki and was approved by the Ethics Committee of the Kyorin University School of Medicine. Written informed consent was obtained from 602 volunteers before participation. Because the number of women was insufficient for classification into groups based on ND2-237 Leu/Met genotype and green tea consumption, women were excluded. Due to glomerular hyperfiltration in early diabetes [30], diabetic patients undergoing treatment were also excluded. Thus, 406 men were enrolled in the study. Twelve individuals with unclear data were also excluded. Therefore, subjects comprised 394 Japanese men aged 29–76 years.

### Clinical characteristics of subjects

Blood chemical and physical data in the present study were obtained from the results of regular medical check-ups [31]. Kidney function was evaluated by estimated glomerular filtration rate (eGFR), which was calculated using a three-variable Japanese equation: eGFR = 194 × creatinine^-1.094^ × age^-0.287^[32]. Based on the K/DOQI CKD classification [27], we adopted the criterion of an eGFR of <90 ml/min/1.73 m^2^ for the mild reduction of eGFR. Body mass index (BMI) was defined as the ratio of subject weight (kg) to the square of subject height (m). A survey of green tea consumption, alcohol consumption, habitual smoking and coffee consumption was performed by means of questionnaire. Green tea consumption was classified based on number of cups of green tea per day (≤1 cup per day; 2–3 cups per day; 4–5 cups per day; and ≥6 cups per day). Alcohol consumption was classified based on drinking frequency (daily drinkers; occasional drinkers, including those who drink several times per week or per month; and non- or ex-drinkers). Smoking status was classified based on number of cigarettes smoked per day (non- or ex-smokers; 1–20 cigarettes smoked per day; and >20 cigarettes smoked per day). Coffee consumption was classified based on number of cups of coffee per day (≤1 cup per day; 2–3 cups per day; and ≥4 cups per day). Subjects were also classified as taking or not taking antihypertensive medication.

### Genotyping

DNA was extracted from white blood cells using the DNA Extractor WB kit (Wako Pure Chemical Industries, Osaka, Japan). ND2-237 Leu/Met polymorphism was detected by polymerase chain reaction (PCR) and digestion with *Alu*I restriction enzyme. The sequence of primers was: forward 5’-CTTAGCATACTCCTCAATTACCC-3’; and reverse 5’-GTGAATTCTTCGATAATGGCCCA-3’. PCR was performed with 50 ng genomic DNA in buffer containing 0.2 μmol/l of each primer, 1.25 mmol/l dNTPs, 1.5 mmol/l MgCl_2_ and 1 U of Taq DNA polymerase. After an initial denaturation at 94°C for 5 min, PCR was conducted through 40 cycles in the following steps: denaturation at 94°C for 30 s, annealing at 60°C for 60 s and polymerase extension at 72°C for 90 s. After cycling, a final extension at 72°C for 10 min was performed. PCR products were digested with *Alu*I restriction enzyme (Nippon Gene, Tokyo, Japan) at 37°C overnight, and were electrophoresed in 1.5% agarose gels stained with ethidium bromide for visualization under ultraviolet light. The absence of an *Alu*I site was designated as ND2-237Met, and the presence of this restriction site was designated as ND2-237Leu.

### Statistical analyses

Statistical analyses were performed using SAS statistical software version 9.2 for Windows. Multiple logistic regression analysis was used to calculate odds ratios (ORs) for the reduction of eGFR (<90 ml/min/1.73 m^2^). For multiple logistic regression analysis and analysis of covariance, alcohol consumption (non- or ex-drinkers = 0, occasional drinkers, including those who drink several times per week or per month = 1, daily drinkers = 2), coffee consumption (≤1 cup per day = 1, 2–3 cups per day = 2, ≥4 cups per day = 3), habitual smoking (non- or ex-smokers = 0, 1–20 cigarettes smoked per day = 1, >20 cigarettes smoked per day = 2), and antihypertensive medication use (no use of antihypertensive = 0, use of antihypertensive = 1) were numerically coded. Differences with P values of less than 0.05 were considered to be statistically significant.

## Abbreviations

BMI: Body mass index; CI: Confidence interval; CKD: Chronic kidney disease; eGFR: Estimated glomerular filtration rate; GTE: Green tea extract; Leu: Leucine; K/DOQI: Kidney disease outcomes quality initiative; Met: Methionine; ND2: NADH dehydrogenase subunit-2; OR: Odds ratio; PCR: Polymerase chain reaction.

## Competing interests

The authors declare that they have no competing interests.

## Authors’ contributions

AK designed the study, carried out the epidemiological survey, carried out genotyping, analyzed the data, and drafted the manuscript; MI collected the samples; NM assisted with genotyping; KK and MY carried out the epidemiological survey; TO, HO, TS, HN and HH assisted in data analysis and helped with interpreting the results; YT designed the study and carried out the epidemiological survey. All authors have read and approved the final manuscript.
